# Inducible nitric oxide synthase (iNOS) in muscle wasting syndrome, sarcopenia, and cachexia

**DOI:** 10.18632/aging.100358

**Published:** 2011-08-07

**Authors:** Derek T. Hall, Jennifer F. Ma, Sergio Di Marco, Imed-Eddine Gallouzi

**Affiliations:** ^1^ McGill University, Biochemistry Department, Goodman Cancer Center, Montreal, Canada

## Abstract

Muscle atrophy—also known as muscle wasting—is a debilitating syndrome that slowly develops with age (sarcopenia) or rapidly appears at the late stages of deadly diseases such as cancer, AIDS, and sepsis (cachexia). Despite the prevalence and the drastic detrimental effects of these two syndromes, there are currently no widely used, effective treatment options for those suffering from muscle wasting. In an attempt to identify potential therapeutic targets, the molecular mechanisms of sarcopenia and cachexia have begun to be elucidated. Growing evidence suggests that inflammatory cytokines may play an important role in the pathology of both syndromes. As one of the key cytokines involved in both sarcopenic and cachectic muscle wasting, tumor necrosis factor α (TNFα) and its downstream effectors provide an enticing target for pharmacological intervention. However, to date, no drugs targeting the TNFα signaling pathway have been successful as a remedial option for the treatment of muscle wasting. Thus, there is a need to identify new effectors in this important pathway that might prove to be more efficacious targets. Inducible nitric oxide synthase (iNOS) has recently been shown to be an important mediator of TNFα-induced cachectic muscle loss, and studies suggest that it may also play a role in sarcopenia. In addition, investigations into the mechanism of iNOS-mediated muscle loss have begun to reveal potential therapeutic strategies. In this review, we will highlight the potential for targeting the iNOS/NO pathway in the treatment of muscle loss and discuss its functional relevance in sarcopenia and cachexia.

## I - INTRODUCTION

**Figure 1:**
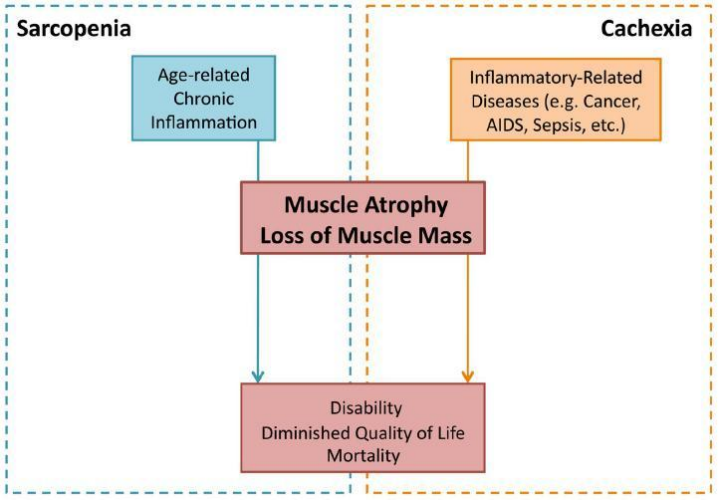
Inflammatory-induced sarcopenia vs. cachexia Sarcopenia and cachexia represent two distinct diseased states, though both can result from an imbalance in the body's inflammatory mechanisms. Whereas sarcopenia (blue) results from chronic inflammation associated with age, cachexia (orange) results from inflammation associated with a primary disease (e.g. cancer, AIDS, and sepsis). Although resulting from different overlying conditions, both sarcopenia and cachexia result in muscle atrophy and loss. The dramatic loss of skeletal muscle tissue, occurring gradually in sarcopenia and acutely in cachexia, leads to disability and increased mortality.

**Figure 2:**
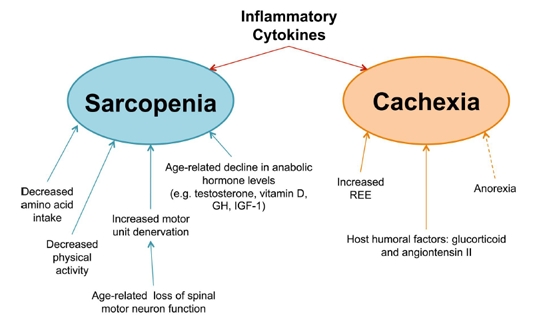
Underlying mechanisms involved in muscle wasting diseases Despite the fact that both diseases result in muscle wasting, the underlying causes of sarcopenia (blue) and cachexia (orange) are distinct. Sarcopenia arises from a multitude of factors, including [1] decreased amino acid intake, [2] diminished physical activity, [3] loss of motor neurons with age, and [4] a decline in anabolic stimulating hormones. Cachexia, in contrast, results from the physiological changes that occur during the progression of other chronic inflammatory illnesses. In cancer, the REE (Resting Energy Expenditure) is known to increase, pushing the overall energy state towards a negative energy balance. This effect is further exacerbated (dashed arrow) by anorexia, which, although not a direct cause of cachectic muscle loss, often accompanies cachexia and contributes towards the overall negative energy balance. Finally, several host humoral factors, such as glucocorticoids and angiotensin II, are known to induce muscle wasting, affecting the overall metabolic state by either by augmenting catabolism, decreasing anabolism, or both. Furthermore, the tumor factor PIF (Proteolysis Inducing Factor) has also been implicated in murine models of cancer cachexia, though its role in human cachexia has yet to be confirmed. In addition to the above factors, inflammatory cytokines are believed to play a key role in the pathology of both sarcopenia and cachexia. As a uniquely common cause of both diseased states, inflammatory cytokines represent an enticing target for the development of drug therapies.

Muscle wasting is a serious affliction commonly found in aging individuals. It results from the combined effects of muscle atrophy as well as muscle cell death, leading to an overall loss of muscle mass and a decrease in muscle strength [1, 2]. The results of muscle wasting are often debilitating and are associated with an increased risk of mortality. In the elderly population, muscle wasting may be found in both acute (cachectic) and chronic (sarcopenic) forms. These two diseased states, though highly interconnected, represent two distinct conditions. Whereas cachexia is only found to develop in the presence of an overlying inflammatory condition, sarcopenia is an age-dependent geriatric syndrome that can develop in the absence of any other apparent pre-existing conditions (Figure 1) [1, 2]. Sarcopenia is associated with a gradual loss of muscle, in contrast to the rapid atrophy associated with cachexia [1]. In some patients, cachexia may lead to the onset of sarcopenia, inducing a state known as cachexia-related sarcopenia [1, 3]. Furthermore, there may be differences in the underlying molecular mechanisms of the two disease states. For example, whereas the importance of ubiquitin-mediated degradation is well established in cachexia, there is conflicting evidence for its role in sarcopenia, suggesting that the proteasomal degradation pathway may play a lesser role in age-related muscle wasting [4]. The existence of sarcopenia in the absence of a primary trigger, as well as the more gradual muscle atrophy that is not associated with an upregulation in ubiquitin-mediated degradation, distinguishes it from cachexia. However, the ability of cachexia to induce sarcopenia underscores the potentially overlapping molecular mechanisms of the two syndromes. Both result in similar changes in the overall metabolic state of muscle fibers, leading to atrophy, and the molecular mechanisms leading to this state may, in fact, share certain common pathways [1, 5]. Indeed, studies have implicated inflammatory cytokines as important humoral factors in the pathology of both sarcopenic and cachectic muscle wasting (Figure 2).

In this review we will briefly describe the molecular mechanisms that lead to both cachectic and sarcopenic muscle wasting, focusing on the shared role of inflammatory cytokines in both syndromes. Specifically, we will highlight the importance of cytokines, such as tumor necrosis factor α (TNFα), as a mediator of muscle wasting and as a therapeutic target for the treatment of both sarcopenia and cachexia. Although recent attempts to target key players in the TNFα signaling pathway have proven ineffective [6-10], several studies have indicated that downstream players could represent better targets to combat muscle wasting. One of these players is the inducible nitric oxide synthase (iNOS) enzyme, which is produced by many cell types, including muscle fiber, in response to TNFα leading to nitric oxide (NO) production and muscle wasting [11, 12]. Growing evidence suggests that the iNOS/NO pathway is involved in both sarcopenia and cachexia-induced muscle wasting [11-14]. Here, we will summarize the current knowledge of the role of this pathway in muscle atrophy and emphasize its potential as a target for future drug development.

## II - SARCOPENIA

The term sarcopenia—a combination of the Greek words sarx, meaning “flesh”, and penia, meaing “loss”—was first coined by Dr. Rosenberg to describe the phenomenon whereby aging individuals would exhibit a gradual loss of muscle mass [15]. Over the years, several clinical definitions of sarcopenia have been proposed [2, 3, 16]. The most recent one defines sarcopenia as the “age-associated loss of skeletal muscle mass and function” [3]. This loss of muscle is considered one of the most dramatic effects of aging on the quality of life of aged individuals [15]. Sarcopenia is a highly prevalent syndrome affecting a large portion of the geriatric population. It is estimated that 25% of individuals over the age of 64 suffer from sarcopenia, and that percentage is doubled in individuals over the age of 80 [17, 18]. Starting at age 30, muscle mass begins to decline at an average rate of 1-2% per year [19]. This gradual loss of muscle eventually leads to increased frailty, resulting in disability and a dramatic increase in the rate of mortality [20]. Currently, the most effective way to prevent sarcopenic muscle loss is through strength training exercise combined with or without amino acid nutritional supplementation [21-23]. However, keeping to a regimented, intensive exercise program may be too difficult or undesirable for many elderly patients [23]. Therefore, it is still important to develop drug-therapies for the treatment of sarcopenia. To date, pharmacological therapies for sarcopenia have been met with limited success [21, 23]. Thus, it is important to elucidate the molecular mechanism of sarcopenia in order to identify new therapeutic targets.

### A - Causes of sarcopenia

Several factors associated with aging are believed to contribute to the onset and progression of sarcopenia (Figure 2). Decreased physical activity, common among aged individuals, has been identified as a risk factor for the development of sarcopenia [24]. Additionally, a lack of adequate amino acid content in the diets of most elderly individuals, compounded by the inherent undernutrition associated with age, is believed to lead to a reduction in anabolic activity that likely contributes to the progressive loss of muscle mass [25, 26]. However, sarcopenia is also associated with obesity and insulin resistance [26, 27]. It has been suggested that the hyperactivity of energy-sensing pathways (such as mTOR) induces muscle loss as a result of a negative feedback loop that causes secondary resistance to growth stimuli [28]. This may explain why caloric restriction, without causing malnutrition, prevents sarcopenic muscle wasting in rhesus monkeys [29]. Besides these life-style factors, other biological mechanisms also play a role in the pathology of sarcopenia [13, 24, 30-40]. The progressive decline of motor neuron function during aging leads to denervation of muscle fibers, which can result in muscle mass loss (Figure 2) [32, 33]. It has also been found that declining hormone levels may play a role in age-related muscle atrophy. In men, low testosterone and vitamin D levels are considered risk factors for the development of sarcopenia [24]. Furthermore, there is an age-related decline in serum levels of growth hormone (GH) and insulin-like growth factor-1 (IGF-1). Loss of these anabolic-stimulating hormones may contribute to the progression of muscle atrophy (Figure 2) [35, 36]. Another known cause of sarcopenia is the age-related increase in proinflammatory cytokines, a process known as inflamm-aging [41]. The proinflammatory state affects the metabolic balance in muscle fibers, leading to muscle atrophy and apoptosis (Figure 2) [42].

### B - Role of inflammatory cytokines in sarcopenia

Several studies have implicated elevated levels of two cytokines, interleukin-6 (IL-6) and TNFα, in the development of sarcopenia. Studies of IL-6 plasma levels in the elderly community found that individuals with higher circulating IL-6 levels were more likely to suffer from fatigue and disability, suggesting that IL-6 may be involved in the age-related decline in muscle function [37, 38]. IL-6 was later found to correlate with lower muscle mass and strength, further implicating it in the progression of sarcopenia [39]. In the same study, TNFα plasma concentration was also found to correlate with lower muscle mass and strength, suggesting that TNFα may be another humoral mediator of muscle atrophy in the aged population [39]. Indeed, in their clinical study of 2,177 men and women, Schaap *et al.* found a correlation between increased serum levels of TNFα and decreased muscle mass and strength over a 5-year period [43]. TNFα is believed to induce muscle cell apoptosis, which may partially account for this geriatric muscle loss [44]. The increase of IL-6 and TNFα serum levels is indicative of an over-all proinflammatory state that develops during the aging process. The activation of the inflamm-aging process is believed to be the result of an upregulation of the transcription factor NF-κB, an important regulator of the innate immune response [42]. This constitutive and elevated activity of NF-κB associated with aging has been suggested to be one of the underlying causes of sarcopenia [45, 46]. As an important mediator of NF-κB induced muscle wasting [12, 47], it is likely that iNOS plays an important role in the pathology of sarcopenia. Indeed, an *in vivo* study comparing the muscle tissue of young and old mice found that iNOS was upregulated in elderly murine muscle, implicating iNOS as a key effecter of sarcopenic muscle loss [13].

## III - CACHEXIA

Cachexia is a fatal syndrome that develops in patients with chronic inflammatory conditions such as cancer, AIDS, and sepsis [48, 49]. Cachexia is characterized by a severe wasting (up to 75%) of skeletal muscle tissue [50]. This dramatic loss of muscle mass leads to loss of motor function, an overall decrease in the quality of life, and a reduced survival rate (Figure 1) [49]. It has been estimated that 2% of the general population is afflicted with early-stage cachexia (defined as weight loss in association with a chronic disease). Of these 2%, it is unknown how many progress to late stage cachexia [51]. However, it is important to note, that cachexia mostly affects patients with chronic diseases. Within this subpopulation, the prevalence of early stage cachexia can reach as high as 36%, as is the case for chronic obstructive pulmonary disease (COPD) [51]. It has been shown that up to 22% of all cancer-related deaths are directly caused by cachexia, rather than the primary malignancy [52]. Death is frequently caused by extensive wasting of the respiratory muscles, leading to hypostatic pneumonia [53, 54]. Cachexia is often accompanied by loss of adipose tissue, anemia, and anorexia, all of which further contribute to its detrimental effects [48, 49]. Despite the importance of muscle wasting syndrome in the pathology of several prevalent diseases, very few treatment options exist for patients with cachexia.

### A - Causes of cachexia

One of the main characteristics of cachexia-related muscle atrophy is that, unlike starvation-induced atrophy, it cannot be reversed with nutritional supplements [48, 55, 56]. This indicates that complex metabolic changes have occurred in patients undergoing muscle wasting. In an attempt to better understand the disease, and to identify potential drug targets for future therapy, many of the underlying causes of cachexia are currently under investigation (Figure 2). Changes in the body's energy balance are believed to contribute to the overall catabolic state found in cachexia. Although not believed to be a direct cause of cachectic muscle loss, anorexia often accompanies cachexia and contributes to the onset of this syndrome [48, 57]. Moreover, the increase in the resting energy expenditure (REE) that is often found in cancer patients may contribute to the overall decline in the energy balance of skeletal muscle tissues [48]. In addition to the general changes in the metabolic energy state, several humoral factors are also believed to trigger muscle atrophy and loss. Proteolysis Inducing Factor (PIF), glucocorticoids, and angiotensin II have all been implicated in the progression of cancer cachexia [48]. Furthermore, it is believed that an increase in circulating inflammatory cytokine levels is one of the main causes of cachexia. Cytokines that have been implicated in the development of cachexia include IL-6, IL-1, TNFα, and interferon-gamma (IFN-γ) [49, 58].

### B - The role of IL-6 and IL-1 in cachexia

IL-6 has been connected to cachexia by both clinical and *in vivo* studies. An investigation into the levels of IL-6 in terminally ill cachectic cancer patients found that IL-6 levels were dramatically elevated one week prior to death [59]. A study of APC^Min/+^ mice—an established murine model for cancer cachexia in which a germline mutation in the adenomatous polyposis coli (APC) gene results in the development of colon cancer [60]—found a severe wasting of the gastrocnemius muscle that correlated with a 10-fold increase in circulating IL-6 levels when compared to wild-type mice [61]. Furthermore, the genetic ablation of IL-6 in these mice prevented the onset of muscle wasting symptoms. The cachectic symptoms were rescued when recombinant IL-6 was over-expressed using a plasmid vector in the IL-6 -/- mice, confirming that IL-6 was necessary for the development of muscle wasting [61]. Interestingly, IL-6 over-expression did not induce muscle wasting in non-tumor bearing mice, indicating that IL-6 may cause muscle wasting indirectly by increasing tumor burden [48, 61]. IL-6 has also been shown to increase the expression and activity of cathepsins (B and L) and ubiquitin, leading to the activation of the lysosomal and proteasomal proteolytic cleavage pathways, respectively [62, 63]. IL-6 signaling involves the STAT-3 pathway, but it is unclear whether this pathway plays a role in IL-6-induced muscle wasting [61]. The effects of IL-6 *in vivo* may be augmented by the secretion of IL-1, which has been shown to increase the expression of IL-6 by colon-26 carcinoma cells [64, 65]. IL-1 has also been implicated in other models of cachexia. Administering IL-1 to rats was found to induce wasting in the peripheral muscles, and IL-1 receptor antagonists prevent sepsis-induced cachexia in rats by recovering protein synthesis [66, 67]. These results show that IL-1 is both sufficient and necessary for the induction of cachexia in rat models, but the role of IL-1 in human cachexia remains unclear.

### C - The role of TNFα/IFN-γ in cachexia

Several animal models for cachexia have shown that TNFα is one of the main cytokines that triggers muscle wasting. Mice implanted with Chinese hamster ovary (CHO) cells transfected with a human TNFα vector develop cachexia and die sooner than mice implanted with CHO cells transfected with a control vector alone [68]. Furthermore, transplantation of Lewis lung carcinoma cells into transgenic mice expressing a soluble TNFα receptor protein showed reduced muscle wasting, even though TNFα serum levels were found to remain constant. This demonstrated that TNFα was necessary for the development of cachexia in this model [69]. TNFα is known to mediate its effects through the transcription factor NF-κB, which is responsible for the regulation of a wide variety of genes [70]. Two of the main mechanisms by which NF-κB induces muscle wasting are through the upregulation of the ubiquitin-proteasome pathway (due to the increased expression of the muscle specific ubiquitin-ligase MuRF1) and through an increase in the expression of iNOS, leading to oxidative stress [10-12, 71, 72]. Treatment with TNFα alone, however, is not always sufficient to induce a major cachectic response. It has been shown that TNFα when administered with the cytokine IFN-γ, triggers a more pronounced cachectic response than if it was administered alone [72, 73]. IFN-γ was first identified as an important humoral factor in the development of cachexia by Matthys *et al.*, who showed that injecting mice with IFN-γ-producing CHO cells induced muscle wasting. The development of cachexia in mice was dependent on the presence of a tumor, indicating that IFN-γ likely mediates its cachectic effect in concert with other cytokines, such as TNFα [74].

### D - The importance of TNFα in human cachexia

Although the role of the TNFα signaling pathway in cachectic muscle wasting has been clearly demonstrated both *in vitro* and *in vivo* [68, 69, 75], its role in human cachexia, and by extension its potential as a therapeutic target, was previously uncertain [48, 58]. However, recent evidence has clearly shown that the TNFα pathway is a significant part of human cachexia pathology. In a study of skeletal muscle from cancer and AIDS patients suffering from cachexia, Ramamoorthy *et al.* analyzed the rectus abdominis and vastus lateralis muscles for the expression of TNFα, iNOS, and several proteins important for muscle differentiation and maintenance, including Jun-D, myogenin, myosin, and CKM (muscle creatine kinase). They found that patients with cachexia had consistently higher levels of TNFα mRNA and protein. Furthermore, they found that the level of active forms of the TNFα receptor was significantly increased in patients suffering from cachexia. In addition, the expression of both iNOS mRNA and protein was found to be upregulated in the muscle tissue of cachectic patients, suggesting a role for the iNOS/NO pathway in TNFα-induced muscle wasting in humans [14]. These results clearly indicate that interfering with TNFα-signaling could be an effective therapeutic strategy to combat cachexia.

### E - Targeting the TNFα pathway: several attempts but little success

Several drugs targeting TNFα signaling have been investigated for their potential use in the treatment of cachexia. Unfortunately, they have all proven to be ineffective therapeutics. Thalidomide, a drug that both augments TNFα mRNA degradation and inhibits NF-κB [50], was found to be successful in preventing the loss of lean body weight in cachectic patients, but failed to improve quality of life and survival rates [6, 7]. In addition, thalidomide has been associated with severe side effects, such as teratogenesis, deep vein thrombosis, and peripheral neuropathy [6, 7]. Two other drugs, Etanercept (a dimeric fusion protein commonly used for the treatment of rhematoid arthritis) and infliximab (a monoclonal antibody against TNFα), have also been met with limited success in clinical trials, showing no improvement in cancer patients suffering from cachexia [8, 9]. To our knowledge, there are no drugs that target TNFα currently in development for the treatment of sarcopenia. Furthermore, the use of pharmacological intervention for the treatment of sarcopenia has also been met with limited success [21, 23]. The ineffectiveness of these pharmacological agents in both sarcopenia and cachexia underscores the need for the identification of new drug targets for the treatment of muscle wasting.

Given the ineffectiveness of targeting TNFα directly, researchers began to investigate the therapeutic potential of targeting downstream effectors in the TNFα pathway, such as NF-κB and MuRF1. NF-κB inhibition therapies have shown potential for the treatment of certain inflammatory diseases, as well as for the treatment of cancer [76-78]. However, given its role as a master-regulator of several pathways, prolonged NF-κB inhibition can result in severe side effects such as immunodepression and liver damage [78, 79]. As both sarcopenia and cachexia persist over prolonged periods of time, any treatment option would need to be viable for extended periods. Thus, targeting downstream effecters of NF-κB-induced muscle wasting may be a better therapeutic option. A key mechanism of NF-κB dependent muscle-atrophy involves the activation of the proteasome degradation pathway by the upregulation of the E3-ligase MuRF1 [10]. Although several studies suggest that preventing protein degradation can help to attenuate the onset of muscle wasting, knocking out the MuRF1 gene does not fully protect mice from NF-κB-mediated muscle wasting [10, 48], suggesting that NF-κB induces muscle atrophy by additional mechanisms. Therefore, given the ineffectiveness of current treatment strategies targeting the TNFα-signaling pathway, there is a need to identify novel therapeutic targets. Despite several studies clearly implicating iNOS as an important effecter in the TNFα/NF-κB pathway, to our knowledge, the iNOS/NO pathway has never been targeted for the treatment of muscle wasting.

## IV - INOS/NO: A COMMON EFFECTER PATHWAY FOR BOTH SARCOPENIA AND CACHEXIA

**Figure 3:**
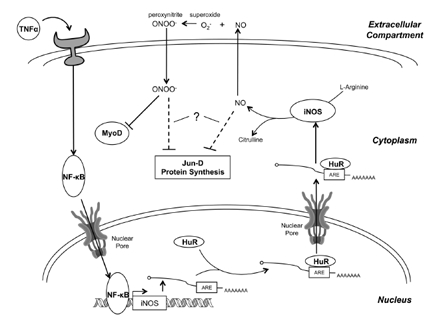
The mechanism of iNOS-induced muscle wasting TNFα, a key proinflammatory cytokine in the induction of muscle wasting, binds to its receptor, activating a signaling pathway that culminates in the activation of NF-κB. NF-κB then enhances the transcription of the iNOS transcript, which is subsequently bound by HuR at an ARE in the 3'-UTR and stabilized. This results in a dramatic increase in iNOS mRNA levels, resulting in enhanced translation of the iNOS protein. iNOS converts L-arginine into citrulline, releasing NO in the process. Several NO-dependent pathways may be responsible for the induction of muscle wasting. First, NO diffuses out of the cell where it combines with superoxide (O_2_^-^) to form peroxynitrite (ONOO^-^). Peroxynitrite then diffuses back into the cell, selectively inhibiting MyoD, an important myogenic transcription factor, at the post-transcriptional level. Loss of MyoD leads to a reduction in MyHC expression, compromising the integrity of the myofibrillar protein complex. Second, NO-production leads to the oxidative modification of Jun-D, which, together with myogenin, regulates several key skeletal muscle-specific proteins, like CKM. Finally, NO-production may inhibit protein synthesis by inhibiting mTOR signaling and by increasing eIF2α and eEF2 phosphorylation, though the mechanism by which this occurs is uncertain. It is also unclear whether NO causes these last two effects directly, or through the formation of peroxynitrite.

As described above, two of the main mechanisms by which TNFα-induced NF-κB activation triggers muscle wasting are through an upregulation of the ubiquitin-proteasome pathway by the increased expression of MuRF1, as well as through an increase in the expression of iNOS, leading to oxidative stress [10, 11, 71, 72]. While, as described above, the TNFα-mediated activation of the proteasome has received a lot of attention in the recent years, specifically as a potential target for therapy against muscle wasting, the implication of the iNOS/NO pathway remained neglected despite several studies linking it to the TNFα-induced muscle atrophy [11, 12, 14]. iNOS converts L-arginine to citrulline, releasing NO in the process. Under certain conditions, NO reacts with superoxide anions (O_2_^-^) to form the toxic molecule peroxynitrite (ONOO^-^), leading to oxidative stress and muscle fiber loss (Figure 3) [12, 80]. Although the detailed mechanism of how NO-induced stress leads to muscle wasting remains to be elucidated, the production of NO, and the subsequent formation of peroxynitrite, has been shown to decrease mRNA levels of MyoD—an important transcription factor involved in myogenesis and maintenance of skeletal muscle [12, 73].

Furthermore, NO generated in response to both endotoxin and IFN- γ has been found to inhibit general translation, leading to decreased protein synthesis in muscle fibers [81].

### A - The iNOS/NO pathway as a promoter of muscle wasting

As previously mentioned, the role of the ubiquitin-mediated degradation pathway in sarcopenia is unclear. However, there is growing evidence for the role of iNOS-induced oxidative stress in both sarcopenic and cachectic muscle wasting. Indeed, several studies have found an increase in the levels of protein nitration with age, suggesting an elevated level of nitric oxide and peroxynitrite production [82-85]. An *in vivo* study evaluating the levels of iNOS expression in old and young mice found a marked increase in the levels of iNOS protein in older mice when compared to their younger counterparts. This increase in iNOS expression was correlated with an age-related increase in caspase-2 and JNK signaling activity, suggesting that the elevated iNOS expression may be involved in age-induced skeletal muscle apoptosis [13]. Several *in vitro* and *in vivo* studies have implicated iNOS in the mechanism of cytokine-induced cachexia [11, 12, 81]. An investigation of the levels of iNOS in the skeletal muscle of COPD patients with low body weight—a marker for the onset of cachexia—found that iNOS expression was elevated [47]. Recently, the induction of iNOS expression by TNFα was demonstrated in human cancer and AIDS patients suffering from cachexia [14]. Thus, given the shared involvement of iNOS in mediating NF-κB signaling in both cachectic and sarcopenic muscle wasting, any therapy that targets the iNOS/NO pathway may prove to be an effective therapeutic strategy in both diseased states.

### B - How does iNOS/NO trigger muscle wasting?

In order to target the iNOS/NO pathway, it is essential that the underlying mechanism be well understood. Recent studies have begun to elucidate the mechanism and have confirmed iNOS as a potential target for the treatment of muscle wasting. Although these studies were carried out in cachectic models of muscle wasting, it is reasonable to assume that similar pathways may be involved in the molecular mechanism of sarcopenia. Indeed, iNOS expression has been shown to increase with age and has been correlated with age-related muscle cell apoptosis [13]. Furthermore, several studies have demonstrated elevated levels of protein nitration with aging, implying an age-dependent increase in the production of peroxynitrite [82-85]. As described below, peroxynitrite production is a key step in the iNOS-mediated muscle wasting pathway (Figure 3), and so together these findings suggest a role for iNOS in several age-related pathologies, including sarcopenia.

#### 1. Inhibition of Jun-D and loss of Muscle Creatine Kinase (CKM)

The first observations connecting TNFα-induced iNOS/NO production to muscle wasting was the discovery that TNFα leads to an upregulation of iNOS, inducing oxidative stress and a loss of Jun-D activity [11]. The study found that injecting mice with CHO cells expressing TNFα -induced muscle wasting and a loss of CKM transcriptional expression. CKM is a kinase that synthesizes ATP from phosphocreatine and is important for the maintenance of ATP reserves and muscle function [86, 87]. Loss of CKM, therefore, is detrimental and provides a molecular indication of the onset of muscle wasting. The decrease in CKM expression was found to be caused by loss of activity of the Jun-D transcription factor. The inhibition of Jun-D was proposed to occur through the post-translational oxidization of its conserved cysteine domain (KCR). The effects of cytokine-induced oxidative stress were found to depend on an increased iNOS expression. This was confirmed by treatment with nitro-L-arginine, an inhibitor of iNOS activity, which was able to prevent the symptoms of muscle wasting, indicating that iNOS was likely the mediator of TNFα-induced oxidative stress [11]. Thus, NO-production by iNOS was shown to be involved in the induction of muscle wasting. The significance of this was later confirmed in human muscle wasting by Ramamoorthy *et al.*, who showed that the skeletal muscle of human cachectic patients had elevated levels of iNOS mRNA and protein [14].

#### 2. Loss of MyoD: The Role of Peroxynitrite

In addition to affecting Jun-D, TNFα treatment was also found to decrease the mRNA levels of the transcription factor MyoD, one of the main promoters of muscle fiber formation [73]. Muscle fibers treated with TNFα were found to have reduced levels of MyoD protein that was associated with a decrease in the mRNA steady-state levels. This inhibition seems to be selective, as other important muscle transcription factors (Myf5 and MEF2D) were unaffected. This effect was also observed in fully differentiated myotubes, and required the addition of IFNγ to potentiate TNFα's effects. The loss of MyoD was dependent on active NF-κB, as indicated by the fact that MyoD mRNA decay could be prevented by over-expressing the NF-κB repressor protein IκBα [73]. Loss of MyoD is known to prevent the differentiation of myoblasts (undifferentiated muscle cells) [73, 88, 89], leading to a reduction in the repair and regeneration capacity of muscle fibers. Furthermore, the TNFα/IFN-γ-induced loss of MyoD has been correlated with the loss of myosin heavy chain (MyHC) in differentiated muscle tubes [73]. The results were also confirmed *in vivo* by injecting mice with a mixture of CHO cells expressing human TNFα and mouse IFN-γ [73]. The selective inhibition of MyoD was later found to result in selective transcriptional down-regulation of MyHC over other myofibrillar proteins, such as tropomyosin, troponin, sarcomeric actin, actinin, and myosin light chain [90]. This apparent selectivity, both for MyoD and subsequently MyHC, suggests that cytokine-induced wasting results not from general loss of muscle protein, but from the specific targeting of key factors. The loss of MyHC in myotubes likely compromises the integrity of myofilaments and could explain the ability of TNFα to reduce muscle strength independently of atrophy and protein loss [91, 92]. Together, these results showed that the loss of MyoD both blocks muscle repair and regeneration and leads to the degeneration of muscle fibers. However, the mechanism by which NF-κB activation caused this was unknown.

MyoD mRNA decay was later found to be caused by the upregulation of iNOS by NF-κB, which was further enhanced by the stabilizing effects of the human antigen protein R (HuR) on the iNOS mRNA transcript (Figure 3) [12]. Microarray analysis of the expression profile of TNFα/IFN-γ treated myotubes showed that iNOS expression was elevated during the first 24 hours of treatment. The increase in iNOS mRNA levels correlated with a corresponding increase in iNOS protein levels and NO production. However, NO on its own was found to be insufficient to induce muscle wasting, as treatment with an NO-donor, which does not produce peroxynitrite, did not induce fiber loss (unpublished data) [12]. Instead, it was found that the formation of peroxynitrite from the reaction of NO with superoxide was responsible for the induction of muscle wasting (Figure 3). Treatment with a peroxynitrite scavenger prevented the loss of fibers induced by cytokine treatment [12]. Thus, loss of MyoD was found to be caused by peroxynitrite through an as of yet unidentified oxidative stress pathway.

#### 3. Posttranscriptional regulation in iNOS/NO-induced muscle wasting

The transcriptional regulation of iNOS by NF-κB is an important part of its cytokine-mediated induction, but it does not account for the full fold-increase in iNOS mRNA expression levels. Cytokine treatment has been found to increase the transcription rate of iNOS only two- to five-fold, whereas iNOS mRNA levels increase up to a 100-fold upon cytokine induction [93, 94]. In accordance with this observation, Di Marco *et al.* found that iNOS mRNA in muscle cells is regulated post-transcriptionally at the level of RNA stability, and that this regulation partially accounts for the increase in iNOS mRNA steady state levels [12]. Previous studies had identified an A/U rich element (ARE) in the 3'-UTR of the human iNOS transcript [95]. AREs are known to destabilize transcripts, resulting in their rapid turnover [96-98]. The RNA-binding protein HuR is known to bind to these AREs, regulating mRNA expression through cellular turnover and nuclear export [99-103]. Often, HuR binding stabilizes the transcript, promoting its expression by extending its half-life. A previous study of the interaction of HuR with human iNOS had shown that HuR binding stabilized the otherwise labile iNOS mRNA [95]. These results were confirmed in an *in vitro* model of cachexia, indicating the importance of HuR-mediated regulation of iNOS during cytokine-induced muscle wasting [12].

#### 4. Inhibition of Protein Synthesis

In addition to the above effects, NO has also been found to induce the phosphorylation of the eukaryotic translation initiation factor 2 (eIF2α) and to inhibit the mTOR pathway, leading to the inhibition of general translation [81, 104]. In a study of the effects of NO on protein translation, Kim *et al.* found that both NO donors and increased iNOS expression were able to reduce protein translation in a variety of cell types [104]. In RAW264.7 murine macrophage cells, this reduction in protein synthesis is caused by the phosphorylation of eIF2α, leading to the inhibition of the 80S ribosomal complex [104]. Further studies are needed to confirm whether this mechanism occurs in muscle cells and to determine whether it plays a role in NO-dependent muscle wasting. In another study, Frost *et al.* showed that cytokine-induced expression of iNOS in mouse muscle cells led to an inhibition of the mTOR pathway, causing a decreased phosphorylation of the downstream targets 4E-BP1 and ribosomal protein S6. The decreased phosphorylation of these factors, in turn, caused a reduction in general translation. In addition, eEF2 phosphorylation was found to increase, suggesting that NO blocks protein translation both at the level of initiation and elongation [81]. They found that this inhibition of general translation could be reversed by treatment with an iNOS inhibitor, confirming the role of iNOS in this cytokine-induced mechanism [81]. These studies demonstrate that prolonged exposure to high doses of NO, as is found in the muscle wasting state, can inhibit general protein synthesis. However, *in vitro* and *in vivo* studies are needed to confirm a role for this pathway in the progression of muscle wasting. Furthermore, it is unclear whether the effects of NO are caused directly or by the production of peroxynitrite, as is seen in NO-mediated MyoD inhibition [12].

#### 5. Future Goals: Expanding the Role of Peroxynitrite

Together, these findings suggest a mechanism whereby the TNFα signaling pathway stimulates the expression of iNOS, both by transcriptional activation by NF-κB and post-transcriptional stabilization by HuR, leading to the production of NO and ultimately peroxynitrite. The peroxynitrite then induces oxidative stress, targeting pathways important for muscle differentiation and maintenance, which results in muscle fiber degeneration. In addition, fiber degeneration may be further enhanced by the apparent NO-mediated inhibition of Jun-D binding activity and protein synthesis (Figure 3). Despite these new insights into the underlying mechanism of the iNOS/NO pathway, several details need to be elucidated. One important question remaining is how peroxynitrite specifically targets only certain myogenic factors. It has been observed that nitrosylation by peroxynitrite proceeds slowly with most biological molecules and that peroxynitrite is therefore a selective oxidant. Nitrosylation is believed to be an important post-translational modification involved in multiple human diseases [87, 105], and may prove to be the mechanism by which peroxynitrite induces muscle atrophy. This apparent selectivity might also explain how peroxynitrite specifically targets MyoD over other myogenic transcription factors. Furthermore, it is possible that peroxynitrite may be responsible for other observed pro-cachectic effects of NO. It has been shown that treatment with antioxidants is able to prevent TNFα-induced reduction in specific tension in isolated, permeabilized mouse muscle, confirming the importance of oxidative modification in the mechanism of TNFα-induced wasting [92]. It is possible that the peroxynitrite-mediated loss of MyoD, and subsequently MyHC, is partially responsible for this loss of specific tension, and so treatment with antioxidants may alleviate TNFα-induced muscle weakening by scavenging superoxide and preventing peroxynitrite formation. It is also unclear whether peroxynitrite is involved in NO-mediated inhibition of Jun-D binding activity and protein translation. More investigation is needed to fully understand the mechanism by which peroxynitrite-induced oxidative stress specifically targets certain vital muscle proteins and triggers muscle atrophy.

### C - Potential Targets in the iNOS/NO pathway to combat muscle wasting

Several pharmacological compounds currently exist for the treatment of sarcopenia and cachexia [21, 23, 48, 49]. However, none of them have been approved for widespread use. Although some of these treatments have shown some promising results, none of them were successfully able to fully reverse the effects of muscle wasting. Consequently, there is a need to identify new targets for the treatment of cachexia that might show equal or greater therapeutic potential.

Several possibilities exist for targeting the iNOS/NO pathway for the treatment of muscle wasting. For example, small molecular inhibitors of the iNOS protein could, conceivably, be able to prevent the detrimental effects of cytokine-induced NO production. In their murine model of cachexia, Buck *et al.* were able to demonstrate that feeding mice injected with TNFα-producing CHO cells with the iNOS-inhibitor nitro-L-arginine was able to attenuate the effects of cachexia [11]. Besides targeting iNOS directly, one could also attempt to inhibit its action by scavenging the peroxynitrite, the oxidative agent effecter of iNOS. Indeed, treatment with the peroxynitrite scavenger FeTPPS has been shown to prevent muscle fiber atrophy and iNOS-mediated MyoD loss [12]. One might also be able to inhibit peroxynitrite by targeting ROS generation in muscle, thereby preventing the formation of peroxynitrite. Furthermore, ROS have been implicated in several models of aging and age-related disease, and so inhibiting their production may prove to particularly effective in combating age-related muscle wasting (sarcopenia) [106-108]. NADPH oxidase is believed to be a significance source of ROS in skeletal muscle [109, 110], and so inhibiting NADPH oxidase may help attenuate muscle wasting symptoms. More intensive investigation is required to fully explore the potential of these options in the clinical treatment of muscle wasting.

## V - CONCLUSION

While mounting evidence supports the implication of the iNOS/NO pathway in muscle wasting, many questions remain unanswered. It is still unknown whether NO or a product of the iNOS/NO pathway compromises muscle integrity by affecting the expression of other key components, besides Jun-D and MyoD, involved in muscle fibers formation, maintenance or both. Moreover, the way by which peroxynitrite leads to the degradation of MyoD mRNA is still elusive. In addition, it is unclear whether peroxynitrite plays a role in NO-mediated inhibition of protein synthesis and Jun-D activity. Further investigation is required to more clearly establish the role of peroxynitrite in muscle wasting. Nevertheless, the ability of peroxynitrite scavengers and antioxidants to prevent muscle wasting symptoms suggests that targeting peroxynitrite may be an efficacious treatment option. Furthermore, given the role of iNOS/NO in other important physiological pathways, such as normal muscle repair and the innate immune response [111, 112], targeting peroxynitrite, either directly or by inhibition of ROS generation, may prove to be a more desirable treatment as it would allow for inhibition of NO-mediated atrophic effects, without affecting NO-mediated beneficial effects. In the end, sarcopenia and cachexia are multifactoral syndromes, and it is unlikely that any one treatment will provide a miracle cure. Instead, a combination of therapies targeting multiple effectors will likely be necessary. To this end, identifying other direct-effectors of muscle wasting as targets for the development of inhibition therapies is an important first step towards the search for a cure for muscle wasting syndrome.
